# Leveraging text skeleton for de-identification of electronic medical records

**DOI:** 10.1186/s12911-018-0598-6

**Published:** 2018-03-22

**Authors:** Yue-Shu Zhao, Kun-Li Zhang, Hong-Chao Ma, Kun Li

**Affiliations:** 1 The Third Affiliated Hospital of Zhengzhou University, Kangfu Road, Zhengzhou, Henan 450072 China; 2 School of Information Engineering, Zhengzhou University, Science Avenue, Zhengzhou, Henan 450001 China

**Keywords:** De-identification, Text skeleton, PHI

## Abstract

**Background:**

De-identification is the first step to use these records for data processing or further medical investigations in electronic medical records. Consequently, a reliable automated de-identification system would be of high value.

**Methods:**

In this paper, a method of combining text skeleton and recurrent neural network is proposed to solve the problem of de-identification. Text skeleton is the general structure of a medical record, which can help neural networks to learn better.

**Results:**

We evaluated our method on three datasets involving two English datasets from i2b2 de-identification challenge and a Chinese dataset we annotated. Empirical results show that the text skeleton based method we proposed can help the network to recognize protected health information.

**Conclusions:**

The comparison between our method and state-of-the-art frameworks indicates that our method achieves high performance on the problem of medical record de-identification.

## Background

**Fig. 1:**
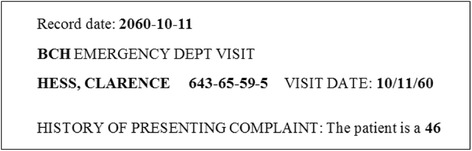
A snippet of an EMR

Electronic Medical Records (EMRs), due to the large amount of information they contain, are valuable resources worth studying. However, because of the large number of Protected Health Information (PHI) existing in EMR, it is difficult for researchers or organizations to obtain these records. Therefore, de-identification of such records is an essential step for using EMRs outside hospitals. Figure 1 shows a sample record with private information, including name, age and record number of patients (highlighted in Fig. 1).

Dorr et al. [1] have evaluated the time cost to manually de-identify narrative text notes (87.2 ± 61 s per note). They concluded that the problem of de-identification was time-consuming. Therefore, a de-identification system is required to automatically detect the personal identifiers. Most of the state-of-the-art systems adopted heuristic or hand-made rules for improving the performance, but the rules are difficult to generalize.

In early 1996, a system named Scrub was proposed by Sweeney [2], through a rule-based approach to hide PHI. In the same year in United States, the Health Insurance Portability and Accountability Act (HIPAA) was passed. 18 categories of information, such as the patients’ names, ID numbers, dates, locations, etc., were defined within the scope of its protection and must be removed from the clinical data before it can be safely de-identified. Since then, many pattern-matching-based and data-driven systems have been introduced [3–6].

To accelerate automated de-identification research, a unified platform to evaluated different systems was firstly provided by the 2006 i2b2 de-identification challenge [7]. In this challenge, eight PHI categories, Patients, Doctors, Hospitals, IDs, Dates, Locations, Phone numbers and Ages, were used to annotate Partner Healthcare data. The competing systems employed rule-based [8] and statistic-based methods. Some researchers considered the challenge as a problem of classification, while others considered it as a sequence labeling problem. These methods include Hidden Markov Models (HMM), Conditional Random Fields (CRFs) [9], Support Vector Machines (SVM) [10], and Decision Trees [11]. Compared with other researches, the results manifested that machine learning-based systems were the best [7].

Along with some recent studies [12], researchers reached an agreement that it is necessary to build a stricter standard than HIPAA. To achieve the goal, the 2014 i2b2 de-identification challenge for longitudinal clinical narratives focused on 25 PHI types, inclusive of 12 types as defined by HIPAA [13, 14]. Some well performing systems submitted to the 2014 i2b2 de-identification track, employed CRFs mixed with dictionaries and regular expressions [15–17].

Li et al. [18] introduced a Stackelberg game to balance risk and utility in EMRs de-identification, they believe their approach is a clear example of risk management approaches to medical data de-identification. Dernoncourt et al. [19] introduced the first de-identification system based on artificial neural network (ANN) and achieved state-of-the-art results on two English datasets.

In this paper, we propose a novel method, which has strong generalization ability, to figure out the de-identification challenge. The method combines text skeleton (TS) and recurrent neural network (RNN) to identify private information in EMRs. The framework, without any structure changed, does well on 2006 i2b2 de-identification challenge, 2014 i2b2 de-identification challenge and a Chinese EMRs dataset annotated by ourselves. The experimental results show that our method is competitive and outperforms the state-of-the-art frameworks at binary token-level. Specifically, the performance on two different i2b2 datasets as well as the Chinese dataset demonstrated an F-score of about 0.98 consistently.

## Methods

### Datasets

We evaluate our model on three datasets: two English datasets from the 2006 i2b2 [7] and the 2014 i2b2 [14] de-identification challenges, one Chinese dataset we annotate by ourselves. The Chinese EMRs come from a maternal and child health-care hospital consisting of 9700 medical records of 485 gravidas. The PHI categories which include dates, IDs, patients, doctors, locations, hospitals and ages are the same as the 2006 i2b2 de-identification dataset. In this work, our dataset is annotated after Chinese word segmentation. Hence the PHIs would not be sliced by mistake. The sizes of the datasets and the distributions of primary PHI categories are presented in Table 1.

**Table 1:** Overview of the datasets

	i2b2–2006	i2b2–2014	Chinese
Number of records	669	1304	9700
Number of tokens	560,852	1,005,582	3,026,944
Number of PHIs	19,498	28,862	48,072
Number of PHI tokens	29,917	38,435	137,496
Vocabulary Size	20,254	41,879	32,265
Percentage of ID	24.6%	3.6%	8.8%
Percentage of DATE	36.4%	43.2%	38.9%
Percentage of HOSPITAL	12.3%	8.0%	2.2%
Percentage of DOCTOR	19.2%	16.6%	14.7%
Percentage of PATIENT	4.7%	7.6%	17.3%
Percentage of AGE	0.1%	6.9%	16.1%

### RNN model

We first present a de-identification system based on RNN as a challenging baseline. RNN is a class of artificial neural network architecture which uses iterative function loops to store information [19]. The long-distance history is stored in a recurrent hidden vector which is dependent on the immediate previous hidden vector. Long Short-Term Memory (LSTM) [20] is one of the most popular variations of RNN. There are several multiplicative gates in LSTM memory cells which can store and access information over long periods of time. Cho et al. proposed Gated Recurrent Unit (GRU) [21] which is a simplification of the LSTM architecture. Cho and his colleagues used neither peephole connections nor output activation functions, but they combined the forget gate and the input gate into a single update gate. They also merged the cell state with hidden state, thus the final model is simpler than standard LSTM models. The GRU architecture can be precisely specified as following equations:

1 $$ {r}_t=\sigma \left({W}_r\cdot \left[{h}_{t-1},{x}_t\right]\right), $$

2 $$ {z}_t=\sigma \left({W}_z\cdot \left[{h}_{t-1},{x}_t\right]\right), $$

3 $$ {h}_t=\left(1-{z}_t\right)\ast {h}_{t-1}+{z}_t\ast \mathit{\tanh}\left(W\cdot \left[{r}_t\ast {h}_{t-1},{x}_t\right]\right), $$

where functions ***σ*** and ***tanh*** are non-linear activation functions. ***r***_***t***_ is reset gate, ***z***_***t***_ is update gate, and ***W*** represent weights.

A bidirectional GRU consists of a forward GRU that moves forward through time beginning from the start of the sequence with another GRU that moves backward through time beginning from the end of the sequence. This structure can provide the output layer with whole past and future context for each point in the input sequence.

**Fig. 2:**
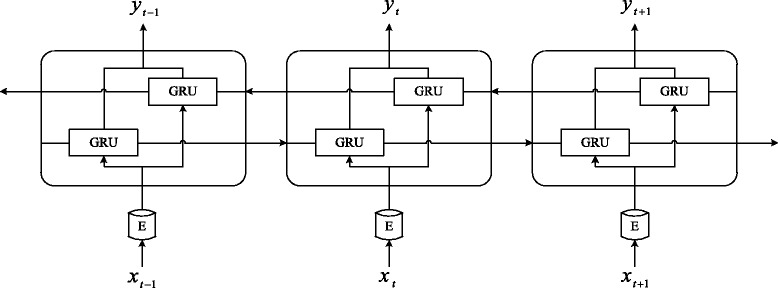
The RNN model for de-identification

The RNN model for de-identification uses the bidirectional GRU, as shown in Fig. 2. *x*_*t*_ is a word of the medical record, *E* is mapping from words to word embeddings, *y*_*t*_ is the predicted label of the *i*-th word.

### Text skeleton

Compared with normal articles or records, there are a mass of short sentences and abbreviations in EMRs. In addition, there are a great number of table-like texts and special writing formats. Therefore, EMRs are a kind of semi-structured text and the efforts to solve the problem of de-identification can also focus on the text structure of the records.

**Fig. 3:**
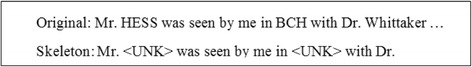
A sample of the text skeleton

Because the forms of EMRs are different from normal traditional text, the skeleton of a record, which helps to privacy information recognition. A statistical approach, proposed by us, extracts the skeletons of records, which reveal the different format and punctuation usage between corpora. Especially, only words that appear more than *t* times during training will be retained and the rest of the words are marked as <UNK> (words in both training set and test set). It should be clearly noted that not only named entities retain but also other words, such as stop words. A text skeleton sample shows in Fig. 3.

We can get various amount of information different skeletons by tuning *t*. In fact, the scope of *t* cannot be easily estimated. Therefore, we propose a method to determine the value of *t* as in Eq. (4). Here *vocabSize* is the vocabulary size of the dataset, *f*_i_ is the number of words whose frequency equal to *i*, maxFreq is the maximum frequency. *r*, a factor which value of is between 0 and 1, which determines the vocabulary size of the skeleton approximately. Thus, appropriate value of *t* can be obtained by tuning *r*.

4 $$ t=\underset{n}{\arg \min}\left|\sum \limits_{i=t}^n{f}_i-r\ast vocabSize\right|, $$

$$ s.\kern0.5em t.\kern0.5em 0<n<\max \kern0.5em Freq, $$

$$ 0<r<1, $$

This approach avoids searching in a large range: by adopting (4) the appropriate *t* often falls in a small range. In different datasets, the best *t* can be smaller than 15 or larger than 100 but the corresponding *r* frequently falls between 0.1 and 0.3.

Sentence is the processed unit, within which the named entities are searched for by many existing systems. If we use the sentence context for the EMRs de-identification, there will be one problem is that there is only one or two words in many sentences. Especially in some extreme cases, a PHI instance is the whole sentence. In order to solve this problem, we concatenate all sentences which come from a record to a unique string, and add a “#RETURN” symbol between every two sentences. Digits whether from training data or from test data are converted into the string DIGIT. For example, “a 46 year old male” we mentioned above will be converted to “a DIGITDIGIT year old male”.

### Combine RNN and text skeleton

**Fig. 4:**
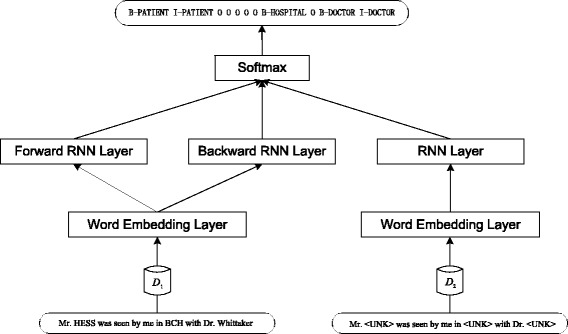
The structure of TS-RNN model

We propose TS-RNN (Text Skeleton- Recurrent Neural Network) by combining RNN with text skeleton. The TS-RNN model is summarized in Fig. 4. There are two branches at the input layer of TS-RNN, which receive original medical record and the text skeleton respectively. Each branch has its own word embedding layer and RNN layer. Through the Softmax layer, each word of the medical record generates a corresponding label. Since the output of the labels are mutually exclusive, we apply Softmax regression after the RNN layer. A label dictionary, which generative process can be combined with the automatic generation of word dictionary, is considered as a necessary condition for determining the Softmax output dimension.

Once the word embeddings have been learned in an unsupervised fashion [22], fine-tuning them during supervised training on the task of interest is possible and has some advantages [23]. Note that there are two dictionaries *D*_1_ and *D*_2_, which are used to map words to the index. Therefore, the two embedding layers are also different. In Fig. 4, the left input branch receives the original text of medical records, and the right branch receives the text skeleton we have introduced. The output labels use BIO tagging scheme to identify PHIs.

A context window is essential for scanning the record, as a record is too long for RNNs. The size of the context window should be selected carefully: a small window size cannot contain enough context information while a large window goes against the learning of model.

### Parameters of the model

Through training subsets of i2b2 datasets, we acquired optimized parameters of the TS-RNN model. The model is trained using Keras [24] with the RMSprop optimizer. Dropout is applied before Softmax for regularization. We used the early-stopping to choice suitable hyperparameters of model on validation set (20% of the training data).

Here are some optimized parameters:

- Dropout: 0.5
- RNN architecture: GRU
- Hidden dimension: 150
- Embedding dimension: 150
- Early-stopping epoch: 8
- Window size: 7
- r: 0.25 (0.1 for 2014 i2b2 dataset and 0.14 for Chinese dataset)

## Results

### PHI identification results on token-level and entity-level

The i2b2 de-identification challenge evaluated at token-level and entity-level, and we used the same way to evaluate our model [7, 14]. Meantime, it is significant to evaluate at binary token-level (PHI token versus non-PHI token). Obviously, the EMRs can be displayed for its completeness. The comparison result is shown in Table 2. Table 2 presents the comparison of F1-scores at entity-level and token-level between the i2b2 submissions and the TS-GRU model. Our goal is to retain non-PHI and to use the complete de-identified EMRs for further medical research.

**Table 2:** Comparison with the i2b2 shared task submissions

	2006 i2b2		2014 i2b2	
	Entity-level	Token-level	Entity-level	Token-level
Submissions	**0.76–0.96**	**0.80–0.97**	0.44–0.93	0.58–0.96
TS-GRU	0.9452	0.9540	0.9344	0.9401

### PHI identification results on different datasets

Here are some the novel models and frameworks for PHI identification. We compare them with our framework, and the results, including the binary token-based precisions, recalls and F1-scores, are shown in Table 3. In the i2b2 2006 de-identification challenge, Wellner et al. [9] achieved the best results, there is no results on the 2014 i2b2 dataset. The Nottingham system [17] was the best system in i2b2 2014 de-identification challenge. Because it’s not publicly available, the system has no results on 2006 i2b2 dataset. MIST [25] is an off-the-shelf program for de-identification and CRF + ANN was proposed by Dernoncourt et al. [19]. CRF is the model based on Conditional Random Field, Bi-LSTM (Bidirectional Long Short-Term Memory) and Bi-GRU (Bidirectional Gated Recurrent Unit) are classic bidirectional RNN models. TS-GRU is the model we proposed in this work.

**Table 3:** Comparison between the state-of-the-art methods and our framework

Model	2006 i2b2			2014 i2b2			Chinese		
	Precision	Recall	F1-score	Precision	Recall	F1-score	Precision	Recall	F1-score
Wellner	0.9870	0.9750	0.9810	–	–	–	–	–	–
Nottingham	–	–	–	0.9900	0.9640	0.9768	–	–	–
MIST	–	–	–	0.9529	0.7569	0.84367	–	–	–
CRF	0.9640	0.9371	0.9504	0.9842	0.9663	0.9752	0.9863	0.9705	0.9783
CRF + ANN	–	–	–	0.9792	0.9784	0.9788	–	–	–
Bi-LSTM	0.9723	0.9656	0.9689	0.9878	0.9389	0.9627	0.9908	0.9584	0.9743
Bi-GRU	0.9871	0.9664	0.9766	0.9750	0.9704	0.9727	0.9898	0.9624	0.9759
TS-GRU	0.9903	0.9855	0.9879	0.9889	0.9723	0.9805	0.9875	0.9719	0.9797

From the binary token-based results we can conclude that the TS-GRU model outperform classical models and previous RNN-based models. Moreover, the TS-GRU model is also competitive at token-level and entity-level. There is an interesting phenomenon: precision of machine learning methods is generally higher than the corresponding recall value but handmade rules can achieve a better recall. Most of medical records are edited on templates, therefore they are semi-structured text. An elaborated regular expression set can work effectively, but these over-complicated rules can match many non-PHIs by mistake.

### Influence on the results by using different factor *r* value

**Fig. 5:**
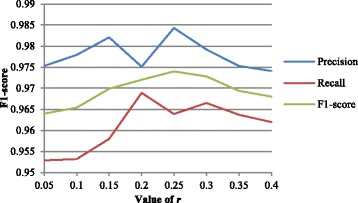
Impact of the value of *r*

Figure 5 shows the impact of the factor *r* on the performance of our model on the 2006 i2b2 dataset. When *r* is between 0.15 and 0.35, the model performs better. If *r* is very small (0.05 or smaller), the dictionary of the skeleton would be small and then the remaining words of a medical record could not reveal the structure of this record. On the other hand, if *r* is very big (bigger than 0.3), the text skeleton will degrade into the original text. The maximum value of *r* in this experiment is 0.4, as the corresponding *t* equals to 2 when *r* is larger than 0.4.

### Influence on the result by setting different window size

**Fig. 6:**
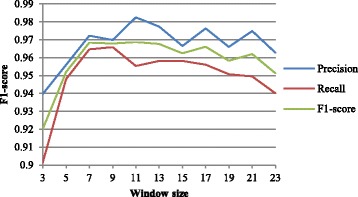
The performance under different window sizes

Figure 6 shows the impact of the size of context window on the performance. With the increasing of the window size, precision, recall and F1-measure increase as well. Yet after 7, F1-score begins to fluctuate slightly around a fixed value.

## Discussion

**Fig. 7:**
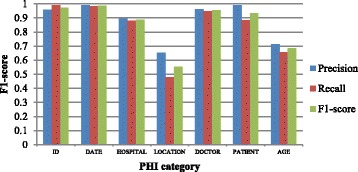
Token-level F1-scores for each PHI category on 2006 i2b2 dataset

Figure 7 shows the token-level F1-scores for all PHI categories on the 2006 i2b2 challenge dataset. Due to the relatively small number of i2b2 datasets in 2006 (the percentages 1% and 0.1% respectively), the LOCATION and AGE categories performed significantly lower than the other categories. Compared with the recall, the precision is higher on most categories except ID. On the category of ID, the recall is about 0.4 higher than the precision. This is due to the fact that all numbers were replaced with the string DIGIT, so the ID numbers can be recognized more easily. In Figs. 6 and 7, the results on the 2014 i2b2 dataset and the Chinese dataset also show that recall is higher than precision on the ID category, which highlights the point.

Table 4 presents the results on the Chinese dataset at entity-level and token-level. In the early stage of our work, the system, based on rule, was established by dictionaries and regular expressions.

**Table 4:** Performance at entity-level and token-level

Model	Entity-level			Token-level		
	Precision	Recall	F1-score	Precision	Recall	F1-score
Rule-based	0.8747	0.9276	0.9003	0.8802	**0.9478**	0.9128
CRF	**0.9815**	0.8972	0.9375	0.9669	0.9236	0.9448
Bi-LSTM	0.9701	0.9235	0.9462	0.9545	0.9027	0.9279
Bi-GRU	0.9665	0.9470	0.9567	0.9592	0.9270	0.9428
TS-GRU	0.9778	**0.9502**	**0.9638**	**0.9777**	0.9447	**0.9609**

**Fig. 8:**
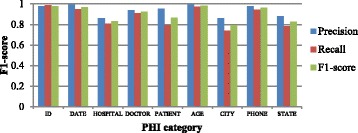
Token-level F1-scores for each PHI category on 2014 i2b2 dataset

Figure 8 shows the token-level F1-scores for all PHI categories on the 2014 i2b2 challenge dataset. The performance of HOSPITAL, CITY and STATE are lower than others because these three categories are quite similar. Sometimes, it’s also hard for humans to classify these words. The performance of AGE is remarkable higher than that in 2006 i2b2 dataset. And because LOCATION is divide into CITY, STATE and so on, the performance of these location names are also superior.

**Fig. 9:**
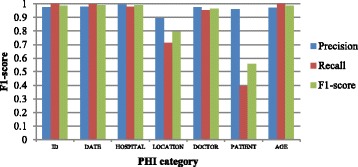
Token-level F1-scores for each PHI category on Chinese dataset

Figure 9 shows the token-level F1-scores for all PHI categories on our own dataset. Compared with recall obtained for other categories, the recall of the PATIENT category is clearly lower. Due to China’s cultural habits, there is no contextual explanation that the patient’s name information does not appear on the record with a uniform format. A more serious problem is that most of the names only appear once in the dataset, and <UNK> information is an important factor in reducing the size of the dictionary. Perhaps it is another reason the names are hard to recognize.

## Conclusions

We proposed a novel de-identification system based on text skeleton and recurrent neural network. Without any structure transform, our method performs well on three datasets mentioned above (two English datasets and a Chinese dataset) at entity-level, token-level and binary token-level. Especially, the results on i2b2 datasets show that the TS-GRU model outperforms classic systems at binary token-level.

Further analysis indicates our method better incorporates the special context in EMRs and is more flexible to different languages than previous systems. Therefore, future research base on TS-RNN will focus on the usage of context and the generation of text skeleton.

## Abbreviations

ANN: Artificial Neural Network
Bi-GRU: Bidirectional Gated Recurrent Unit
Bi-LSTM: Bidirectional Long Short-Term Memory
CRFs: Conditional Random Fields
EMR: Electronic Medical Record
GRU: Gated Recurrent Unit
HIPAA: Health Insurance Portability and Accountability Act
HMM: Hidden Markov Models
LSTM: Long Short-Term Memory
PHI: Protected Health Information
RNN: Recurrent Neural Network
SVM: Support Vector Machines
TS: Text Skeleton
TS-RNN: Text Skeleton- Recurrent Neural Network
